# Unlocking the significant worldwide potential of better waste and resource management for climate mitigation: with particular focus on the Global South

**DOI:** 10.1177/0734242X241262717

**Published:** 2024-07-28

**Authors:** David C Wilson, Johannes Paul, Aditi Ramola, Carlos Silva Filho

**Affiliations:** 1 Environmental and Water Resources Engineering, Department of Civil & Environmental Engineering, Imperial College London, London, UK; 2 Deutsche Gesellschaft für Internationale Zusammenarbeit, Makati City, Philippines; 3 International Solid Waste Association, Rotterdam, Netherlands; 4 Brazilian Institute of Waste Management – iPNRS, Sao Paolo, Brazil

**Keywords:** Municipal solid waste management, climate change mitigation, methane emissions, landfill, open burning, circular economy, developing countries, climate finance

## Abstract

Numbers do matter; the Intergovernmental Panel on Climate Change (IPCC)’s 2010 data that the waste sector is responsible for just 3% of global greenhouse gas (GHG) emissions has led to the misperception that solid waste management (SWM) has little to contribute to climate mitigation. Global efforts to control methane emissions and divert organic waste from landfills had already reduced direct emissions. But end-of-pipe SWM has also been evolving into more circular waste and resource management, with indirect GHG savings from the 3Rs (reduce, reuse, recycle) which IPCC accounts for elsewhere in the economy. The evidence compiled here on both direct emissions and indirect savings demonstrates with *high confidence* that better waste and resource management can make a significant contribution to climate mitigation, and must form a core part of every country’s nationally determined contribution. Even the most advanced countries can still achieve much from the 3Rs. In the Global South, the challenge of extending waste collection to all and stopping open dumping and burning (sustainable development goal 11.6.1), essential to improve public health, can be turned into a huge opportunity. Moving early to divert waste from landfill by separation at source and collecting clean organic and dry recycling fractions, will mitigate global GHG emissions, slash ocean plastics and create decent livelihoods. But this can only happen with targeted climate, plastics and extended producer responsibility finance; and help to local communities to help themselves.

## Introduction

Numbers do matter, and the Intergovernmental Panel on Climate Change (IPCC)’s 2010 data in their 2013 Fifth Assessment Report (AR5) (Blanco et al., 2014) that the waste sector is responsible for around 3% of global greenhouse gas (GHG) emissions has led to a common misperception that waste management has little to contribute to the mitigation of climate heating. To avoid double counting, IPCC partition the economy into sectors, with the ‘waste sector’ confined to end-of-pipe waste management. More than 90% of the waste sector’s direct contribution to climate heating is methane from the anaerobic degradation of organic wastes, both from dumpsites and solid waste landfills but also from wastewater treatment. Methane is a ‘short lived climate forcer’ (SLCF) which has a global warming potential (GWP) around 28 compared to CO_2_ over the standard averaging period of 100 years, but around 86 over the first 20 years after release into the atmosphere (IPCC, 2021; Mar et al., 2022). It has thus received much focus in international efforts to meet the Paris commitment to limit global heating to 1.5°C (Höglund-Isaksson et al., 2020; Ocko et al., 2021; UNEP and CCAC, 2021); the Global Methane Pledge (2021) was launched at COP26 in Glasgow and had 155 participating countries by early 2024. In the AR5 reporting year, 2010, most methane emissions from landfills still came from high income countries, even though most of those countries had already slashed emissions through a combination of collection and treatment of landfill gas and diversion of wastes from landfill (see below; Wilson, 2023).

In addition, over the last 30 years, end-of-pipe solid waste management (SWM) has been evolving into more sustainable ‘waste and resource management’, moving towards a circular economy and even the ultimate ambition of ‘zero waste’, with a focus on realising the 3Rs of reduction, reuse and recycling (Wilson, 2023). Each of the 3Rs can contribute to climate mitigation by displacing GHG emissions elsewhere in the economy: IPCC credits those indirect savings to the upstream sector which put the product or material on the market, or which generated the displaced energy. Even the most advanced high-income countries still have much potential for climate mitigation from the 3Rs.

**Figure 1.**
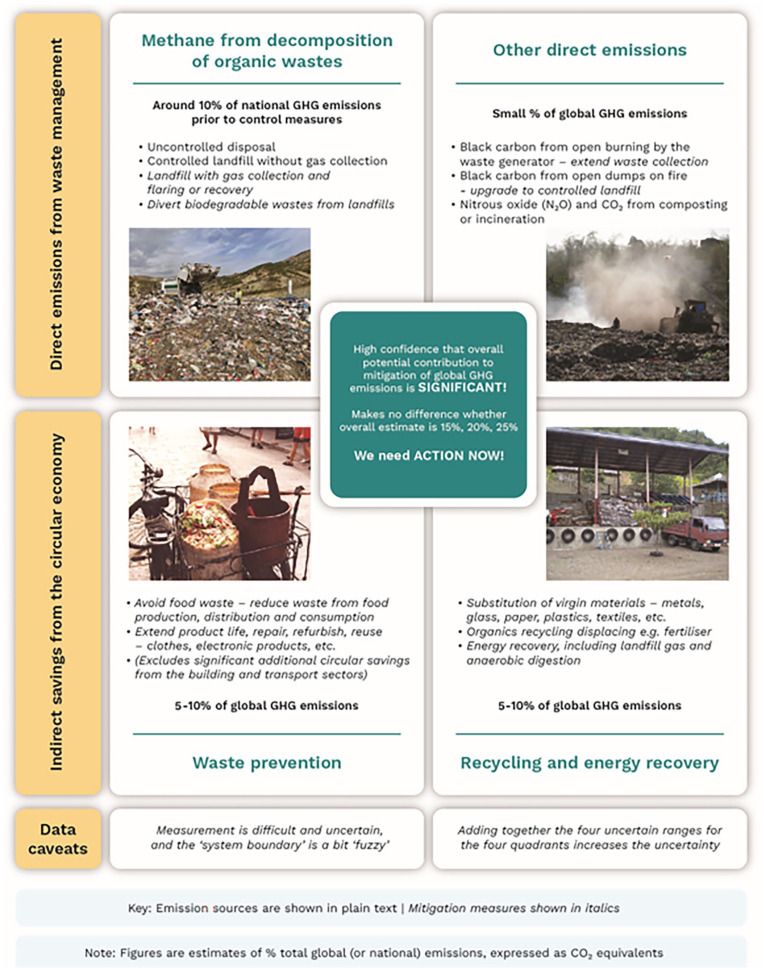
Potential contribution of better waste and resource management to mitigation of global GHG emissions. Source: Infographic © David C Wilson. Photo credits (clockwise from top left): © Brian McCarthy (Albania, 2021 – note the gas well); © Johannes Paul (Philippines, 2010); © Johannes Paul (Philippines, 2011); © David C Wilson (China, 2000). Figure adapted from that used in Wilson et al. (2023). GHG: greenhouse gas.

The purpose of this article is to examine the potential contribution of better (solid) waste and resource management to the mitigation of climate heating; wastewater treatment is beyond the scope. The first two sections examine how (much) direct GHG emissions contribute to climate heating; and indirect GHG savings contribute to climate mitigation. The results are summarised as an infographic in Figure 1. The third section then focuses on the challenges for, and how to unlock the opportunities in, the Global South: how can municipal solid waste (MSW) collection be extended to all citizens, and uncontrolled disposal and open burning phased out, while at the same time mitigating GHG emissions.

This ‘perspectives’ article intends to present a rigorous and well-referenced argument and to contribute at the cutting edge of an important scholarly, societal and political debate of international significance. It has been developed from an earlier, less rigorous article in the trade press (Wilson et al., 2023). Attempting to collate quantitative information on the contribution to total GHG emissions and their mitigation in a country or city, made by direct and indirect GHG emissions attributable to waste and resource management, is both challenging and frustrating. Measurement is difficult and uncertain; each country and city is different, GHG emissions change over time and other sectors take mitigation actions; the results of, for example, life cycle analysis (LCA) are very variable depending on the specific local conditions and the assumptions made (Bisinella et al., 2024; Christensen et al., 2020; Mulya et al., 2022); and the ‘system boundary’ is a bit ‘fuzzy’. So any estimated numbers for the four segments in Figure 1 will always provide ranges and be subject to high uncertainty. Adding such estimates together to provide a total further exacerbates the problem. Nevertheless, our contention is that one can still, using IPCC terminology, have *high confidence* that the overall contribution of better waste and resource management to the mitigation of climate heating is *significant*. So action is needed NOW to ensure that better waste and resource management, and improved resource efficiency, are included as a core part of every country’s nationally determined contribution (NDC).

## How much do direct emissions contribute to climate heating?

### Methane from decomposition of organic wastes

**Figure 2.**
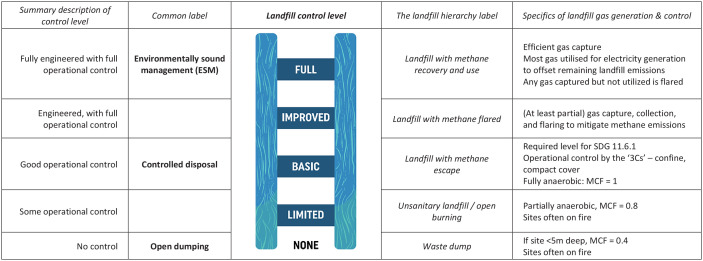
Landfill gas control ladder. The landfill ladder shown here is one example of a control ladder for recovery and disposal, taken from UN-Habitat (2021)’s Waste Wise Cities Tool. The basic level must be met to ‘count’ towards indicator SDG 11.6.1 (controlled disposal). The full control level corresponds to ESM. IPCC in their guidelines for calculating GHG emissions from the waste sector provide default values for the relative methane generation for a fully anaerobic, basic control, controlled landfill as a MCF of 1.0, with lower MCFs for the more aerobic limited control disposal sites and no control dumpsites (IPCC, 2006, 2019). Source: The descriptions in Column 1 for each control level summarise those in UN-Habitat (2021). Ladder figure first published in Whiteman et al. (2021). Figure © Andrew Whiteman and David C Wilson (Graphics: Ecuson Studio). The five-level landfill hierarchy in Column 4 is a subset of an extended version of the EU waste hierarchy, as cited in IPCC’s AR4, waste chapter, Figure 10.16 (Bogner et al., 2007). SDG: sustainable development goal; ESM: environmentally sound management; MCF: methane correction factor; IPCC: Intergovernmental Panel on Climate Change; GHG: greenhouse gas.

IPCC’s AR5 (Blanco et al., 2014) uses 2010 data. The first environmental control legislation was introduced in high income countries from the early 1970s, which gradually increased the level of control over SWM (Wilson, 2023), including for landfills (Figure 2). The early operational controls (the ‘3Cs’ – confine, compact, cover) (Wilson, 2023) resulted in anaerobic controlled disposal sites (the basic control level (Figure 2) now set to count towards sustainable development goal (SDG) indicator 11.6.1), thus *increasing* methane generation. The next steps to improve controls were capture and collection of landfill gas and use of flares to convert methane to CO_2_, which provided significant climate mitigation. Full control or environmentally sound management (ESM) enabled energy recovery from the gas, thus offsetting part of the already mitigated GHG emissions. Following adoption of the UN Framework Convention on Climate Change (UNFCCC) 1992, these environmental controls were supplemented by moves to divert organic wastes from landfills, explicitly to reduce GHG emissions. So by 2010, the estimated 3% direct emissions from the waste sector, as measured by AR5, reflects more than 30 years of effective efforts to mitigate methane emissions from landfill in many high income countries.

Comprehensive data on GHG emissions goes back to 1990, set as the base year under the UNFCCC. The UK is selected as a case study here, partly because 88% of MSW was still disposed of to landfills in 1990. Table 1 shows the evolution of the waste sector’s contributions to total UK GHG emissions from 1990 to 2021; the 1990 baseline already reflected mitigation of landfill emissions due to improved controls with gas collection and either flaring and/or gas recovery.

**Table 1.** Summary of UK GHG emissions national statistics for the waste sector (Mt CO_2_eq year^−1^; rows in italics show percentages).

Sector year	1990	1995	2000	2005	2010	2015	2021
Waste management	72.1	75.2	67.7	52.8	31.5	21.2	18.7
Of which: Waste-water handling	3.1	3.2	2.9	2.6	2.8	2.8	2.8
SWM	69.0	72.0	64.8	50.2	28.7	18.4	15.9
Of which: Landfill	67.4	70.7	63.7	49.0	27.2	16.4	13.6
National grand total	813.4	762.3	725.8	698.7	613.0	510.4	426.5
SWM as % of national total	8.5	9.5	8.9	7.2	4.7	3.6	3.7
Landfill as % of national total	8.3	9.3	8.8	7.0	4.4	3.2	3.2
National GHG savings since 1990	–	51.1	87.6	114.7	200.4	303.0	386.9
Savings from SWM sub-sector	–	−3.0	4.2	18.8	40.3	50.6	53.1
Savings from SWM as % of total savings	–	NA	4.8	16.4	20.1	16.7	13.7

Source: Table 1.2 in UK National Statistics (2022). Table used in Wilson et al. (2023).

NA: not applicable; SWM: solid waste management; GHG: greenhouse gas.

UK landfill GHG emissions remained relatively stable through the 1990s, before sharp reductions from 2000 which reflect the introduction of full ESM controls under the EU Landfill Directive, as well as EU mandated diversion of waste from landfills as an explicit climate mitigation measure. In 1995, landfills alone accounted for 9.3% of total national GHG emissions, reduced to 3.2% by 2021. This sharp fall means that the SWM sector has out-performed other larger sectors, contributing 20% of the overall national mitigation achieved between 1990 and 2010. This illustrates the potential of the SWM sector to provide ‘quick wins’.

By 1990, some other high-income countries had already begun to move proactively away from landfill of MSW, particularly in Japan, and in northern/central European countries where heat from energy recovery incinerators had a ready market for district heating in winter (Wilson, 2023). According to IPCC’s third assessment report (TAR or AR3) (Moomaw et al., 2001), by the mid-1990s, Germany’s MSW landfill emissions still accounted for 3–7% of total GHG emissions. In the United States, 55% of MSW was landfilled in 1997 at sites with at least some gas control, accounting for 4% of national GHG emissions.

Some examples from specific studies in middle- and low-income countries support the hypothesis that the % contribution of SWM declines over time as control over landfills improves and organic wastes are diverted to other management methods. An LCA of three upper middle income countries in 2005/2007 (Dehoust et al., 2010) showed that methane from landfills accounted for 6.8–13.5% of total national CO_2_-eq emissions in Tunisia and 7.2–10.5% in Turkey, with the ranges reflecting assumptions of 50–100% disposal in controlled (anaerobic) landfills without gas control; Mexico showed a range of 6.2–8.0%, reflecting 100% controlled landfills, with gas capture in the range of 100% (lower estimate) to 20%. Other literature examples include Jordan at 10.6% in 2006 (Abu Qdais et al., 2019) and Pakistan at 9–14% in 2016 (Devadoss et al., 2021) – in both cases, most MSW was reported as ‘dumped’. Taken together, all of these examples suggest that, when MSW is collected and disposed of in basic controlled landfills, which meet SDG 11.6.1 but do not yet include gas control (Figure 2), the baseline contribution to national GHG emissions is likely to be around 10%.

The composition of MSW in middle- and low-income is predominantly organic, unlike high-income countries (UNEP and ISWA, 2015); one would thus expect higher methane generation from comparable landfill sites. Results for specific cities do suggest that direct contributions from the waste sector can be even higher than 10% (clearly the % contribution does depend heavily on how much fossil-fuel-intensive industry operates in the city). The waste sector was the second largest in Rio de Janeiro in 2005, accounting for 21% of total GHG emissions, with 14% from solid waste (Loureiro et al., 2013). Two more examples come from the C40 Cities (n.d.) programme to prepare climate action plans by 2020. In Accra (2020), Ghana, the 2015 baseline showed waste as the largest emitting sector at 44%, with SWM contributing 30%, split into 17% controlled landfill, 10% open dumping and 3% open burning and other incineration. In Quezon City (2021), Philippines the waste sector contributed 19% of total emissions in 2016, with 13% from SWM.

### Other direct emissions

Methane is generally considered to account for more than 90% of waste sector GHG emissions. Small contributions are also made by CO_2_ and nitrous oxide (N_2_O) emissions from incineration (Astrup et al., 2009; UNFCCC, 2018) and composting (Nordahl et al., 2023). N_2_O is a powerful SLCF with a GWP compared to CO_2_ of 300.

Discussion here focuses on emissions of black carbon (another powerful SLCF) (Bond et al., 2013) from open burning of waste, which were excluded from IPCC assessment reports up to and including AR5 in 2013 due to a lack of reliable data. Open burning is an important means of household self-management of MSW that is not collected; and less controlled disposal (dump) sites are frequently on fire (Figure 2). Estimating GHG emissions from open burning multiplies together an activity estimate, that is, how much MSW is open burned; an emission factor, that is, the mass of black carbon emitted per kilogram of waste burned; and the GWP for black carbon.

Much work over the last 10 years has focused on improving estimates for each of these. Activity level estimates have ranged from 33 million tonnes per annum (Mtpa) (Bond et al., 2013), to 970 Mtpa (41% of global MSW generation) (Wiedinmyer et al., 2014), to 394 Mtpa (16% of global MSW, split 45:55 between residential and dumpsite burning) (Gómez-Sanabria et al., 2022). The first estimate excludes rural wastes; the second in effect assumes that all waste in low- and middle-income countries is open burned; the third is likely the best available estimate for 2015 MSW data. Measurement of emission factors is difficult, and much early reliance was placed on a few field measurements (Christian et al., 2010). Laboratory experiments showed that most of the black carbon from open burning comes from plastics, specifically PET, polystyrene and fibre (Reyna-Bensusan et al., 2019). The GWP for black carbon was estimated in the range 1200–5100 (Bond et al., 2013); but has been revised to around 800 (IPCC, 2021; Mar et al., 2022).

Based on their measured emission factors and the then current estimates for activity levels (41% of total MSW) and for GWP, Reyna-Bensusan et al. (2019) estimated the contribution of black carbon from the open burning of MSW to global GHG emissions as in the range 2.3–9.7%. Recalculating using the best current estimates of 16% open burning and GWP of 800, with the same emission factors, gives an estimate ~0.7%.

## How much could indirect GHG savings from the 3Rs contribute to climate mitigation?

There is an apparent dichotomy in the IPCC’s discussion of waste and resource management. On the one hand, for accounting purposes, they define the waste sector narrowly as ‘end-of-pipe’ to avoid any double counting (IPCC, 2006). On the other hand, from the beginning they have recognised cross-sector opportunities for savings through the 3Rs – reduce, reuse, recycle.

The second assessment report (SAR or AR2) has little discussion on waste, but does emphasise the (indirect) reduction of GHG emissions from the industrial recycling of metals, glass and paper (Kawasaki et al., 1996). The TAR or AR3 cites that emphasis and sets out both ‘the methane emissions from landfill and the consumer dimension of recycling’ (Moomaw et al., 2001). The fourth assessment report (AR4) stands alone as the only one to give Waste its own chapter (Bogner et al., 2007); that does focus on the ‘narrow’ end-of-pipe definition, but also briefly discusses the 3Rs. In AR5, the main discussion of the waste sector is as an Annex to the Industry chapter; the Executive Summary to the chapter states ‘The most effective option for mitigation in waste management is waste reduction, followed by re-use and recycling and energy recovery (robust evidence, high agreement)’ (Fischedick et al., 2014). This is correct, but also curious: most/all of this mitigation is credited by IPCC to other sectors, not to the waste sector. In the most recent assessment report AR6, waste prevention, minimisation, recycling and management are discussed briefly in the Urban chapter (Lwasa et al., 2022), with more extended discussion in the Industry chapter (Bashmakov et al., 2022); the latter highlights materials efficiency and circular economy (including the 3Rs) as two of the seven key mitigation options.

### Recycling and energy recovery

IPCC’s SAR (AR2) (Kawasaki et al., 1996) emphasised the GHG savings from industrial recycling, documenting a 4- to 5-fold reduction in GHG emissions for secondary rather than primary production of glass, paper, steel and copper, and a 40-fold decrease for aluminium. Later LCAs have broadly confirmed these orders of magnitude, although the net savings from recycling are particularly dependent on the local conditions and assumptions in the life cycle assessment (Bisinella et al., 2024). Steel production accounted for 10% of global 2019 GHG emissions, so increasing recycling has a significant potential for mitigation (Kang et al., 2022). The tonnage of aluminium is much lower, but production accounts for 8% of global electricity use so the mitigation potential is high unless renewable electricity is used in virgin production (Bisinella et al., 2024).

IPCC’s TAR (AR3) discussed the mitigation potential of post-consumer recycling from MSW (Moomaw et al., 2001). They suggested that the then current recycling in the US was reducing national GHG emissions by 2%, and that if recycling rates were to rise to those in the best performing city, Seattle, mitigation would increase to 6% (Ackerman, 2000). Returning to the UK case study, it was estimated that sorting and recycling alone helped avoid 45 MtCO_2_eq emissions in 2018 (ESA, 2021) (9.7% of total national GHG emissions of 465 Mt, see Table 1). Given the average composition of MSW, LCA studies often point to paper recycling as contributing most to GHG mitigation (Christensen et al., 2009, 2020), with significant contributions also from aluminium, ferrous metals, plastics (Gentil et al., 2009) and potentially also textiles.

Energy recovery from waste also provides potential GHG savings, the amount of savings depending on the fuels that are displaced (highest for coal, lowest for renewables). Electricity generation from landfill gas can sometimes turn a landfill site from a GHG emitter into a net savings (Manfredi et al., 2009); the calculation of savings from incineration depends both on whether there is a user for the heat as well as the electricity generated, and on the nature and water content of the biogenic fraction of the waste. The controlled burning of waste of biological origin can be regarded as part of the natural carbon cycle, whereas burning fossil plastics and textiles is not (Astrup et al., 2009).

While LCA studies give widely varying results for the specific benefits of material and energy recovery from MSW (Zhang et al., 2021), they are consistent in showing substantial net benefits (Christensen et al., 2020).

### Waste prevention

IPCC’s AR5 concluded that waste reduction is the most effective mitigation option for waste management (see above; Fischedick et al., 2014). AR6’s industry chapter highlights materials efficiency and circular economy (including the 3Rs) as two of the seven key mitigation options, concluding that ‘(These) are not well understood from a policy perspective and were for a long time neglected in low-GHG industry roadmaps although they may represent significant potential’ (Bashmakov et al., 2022). UN reports have showcased the benefits of incorporating circular economy measures into NDCs or national plans for GHG mitigation (UNDP and UNEP, 2020; UNFCCC, 2018); a roadmap towards circular economy smart NDCs is available (Diaz-Bone et al., 2022); as is a recent review (Yang et al., 2023). UNEP (2021a) co-ordinate a Global Alliance on Circular Economy and Resource Efficiency (GACERE).

Options for the circular economy are now often cited as the ‘9Rs’, of which there are actually 10, arranged into 3 groups: smarter product use and manufacture – R0 Refuse, R1 Rethink and R2 Reduce; extend lifespan of product and its parts – R3 Reuse, R4 Repair, R5 Refurbish, R6 Remanufacture R7 Repurpose; and useful application of materials R8 Recycling, R9 Recovery (including energy recovery) (Morseletto, 2020; Potting et al., 2017). So ‘waste prevention’ can be sub-divided into at least 8 ‘Rs’.

Food waste prevention has received much attention (FAO, 2011), with a specific SDG Target, 12.3, to halve food waste from markets, shops and homes by 2030. The first UNEP (2021b) report monitoring progress using the global food waste index estimated that 931 million tonnes of food, or 17% of global food production, ended up as food waste at the consumer level in 2019, much of which was edible; the split was 61% from households, 26% food service and 13% retail. It has been estimated that 34% of global GHG emissions are from food production (Crippa et al., 2021); so producing food that reaches consumers but goes to waste rather than being eaten accounts for around 6% of global emissions.

Food is not the only product consumers buy in which GHG emissions are embedded. One example is so called fast fashion – both cotton and synthetic fibre production is energy intensive (the textiles and footwear industry account for around 8% of global GHG emissions), so designing clothes and other textile products to last longer and to be easier to repair and reuse would have very significant GHG benefits. Another example is consumer electronic products such as mobile phones and laptops (UNDP and UNEP, 2020).

Looking beyond consumers, the potential for carbon mitigation from materials efficiency, resource substitutions and circular economy measures across the economy is huge. The Circularity Gap report estimates that materials handling and use accounts for 70% of global annual GHG emissions, and that this could be cut in half by circular economy measures, particularly in the buildings, transport and food sectors (Circle Economy Foundation, 2021). Yet, their measure of global circularity, the proportion of materials entering the economy that are secondary rather than virgin, has fallen by 20% in the 5 years since it was first measured, from 9.1% in 2018 to 7.2% in 2023 (Circle Economy Foundation, 2024); it is clear that implementation of the circular economy requires real action, rather than ‘lip service’.

An underlying thread to this article is the confusion caused by different allocations between sectors. A seminal report by the Ellen MacArthur Foundation split the economy into two, with 55% of global GHG emissions associated with energy generation and use, and 45% with product manufacture, particularly food, steel, cement, plastic and aluminium (Ellen MacArthur Foundation, 2019). The Circularity Gap figures above allocate a significant proportion of what is here ‘energy’ to ‘products’, for example that for freight transport. Industry accounts for 21% of global GHG emissions; the savings from circular economy measures including eliminating waste and pollution, and circulating products and materials to retain their embodied energy were estimated by UNFCCC (2018) at 30–50%; and by Ellen MacArthur Foundation (2019) at 40% (i.e. 8% of global emissions). These reports argue that transitioning to a circular economy and curbing GHG emissions are essential to future economic growth. The Circularity Gap Reports look also at human wellbeing, showing that circular economy measures can deliver societal needs with 30% less materials than we currently use, while at the same time providing decent work (Circle Economy Foundation, 2024).

So, any estimates of the range of GHG savings available from recycling and energy recovery, and from waste prevention, will necessarily be uncertain and have fuzzy boundaries. But one can have *high confidence* that they are significant. The estimates shown in Figure 1, which are each 5–10% of global GHG emissions, are quite conservative.

## Focus on the Global South

### The challenge

The world is facing a global waste emergency (UNEP and ISWA, 2015) characterised by a lack of access to MSW collection for 2.7 billion people (UNEP and ISWA 2024; Wilson, 2023) and the alarming statistic that 40% of collected MSW ends up in uncontrolled disposal which often includes open burning (Gomez-Sanabria et al., 2022; Wilson, 2023). This crisis is ongoing and threatens to worsen in low- and lower-middle income countries as populations grow, urbanisation accelerates and waste generation per capita increases with economic development. Without action, many cities in Africa and South Asia are projected to see their waste generation double every 15–20 years (UNEP and ISWA, 2015). To tackle this emergency, it is essential to extend MSW collection to all, while phasing out open dumps and open burning (SDG 11 sustainable cities – indicator 11.6.1).

Tackling this waste emergency will have benefits across many SDGs (UNEP, 2023; Wilson, 2021). Those most impacted by unmanaged or mismanaged wastes are the most vulnerable in society (SDG 1.4 – basic services for all; SDG 10 – reduce inequality). The local benefits often focus on public and individual health (SDG 3) and the environment (SDG 6.3 – improve water quality by eliminating dumping; SDG 12.4 – ESM of all wastes). Global benefits include significant contributions to climate mitigation (SDG 13) and to the prevention of plastics pollution and of ocean plastics (SDG 14.1). An early estimate was that achieving SDG 11.6.1 worldwide would at least halve the mass of plastics reaching the oceans (CIWM and Wasteaid, 2018). Recent modelling has shown a 77% reduction in macroplastics dispersal to the environment and 90% reduction in open burning – resulting in an overall macroplastics pollution reduction of 85% compared to the 2020 baseline (Tanner et al., 2024).

So, what is the potential contribution of better SWM and meeting SDG 11.6.1 in low- and middle-income countries, to mitigation of global GHG emissions? Two recent article s have adapted large climate models and databases to estimate future emissions from the (narrow) municipal solid waste management (MSWM) sector for various scenarios up to 2050 (Gómez-Sanabria et al., 2022). Hoy et al. (2023) modelled how MSWM could meet its portion of the mitigation required to meet the Paris Agreement 1.5°C and 2°C goals or the terms of the Global Methane Pledge (2021). To make the modelling more tractable, they focused on the 43 current highest MSW generating countries in 2020, which contributed 86% of current arisings. The sample was biased to high income countries (14) and the larger middle income countries (13 each upper- and lower-middle income), with just 3 low income countries included. Our question here is, is it reasonable to exclude 130+ countries from the analysis? Some of those are smaller high income countries, but most are in the Global South, including many of the lowest income countries.

To explore this, it is first estimated that that sub-set of the lowest income countries excluded by Hoy et al. (2023) generated (in round numbers) around 10% of global MSW in 2020. How will the generation and management of that waste change by 2050? As noted above, quantities will at least double. Collection coverage is likely <50% (UNEP and ISWA, 2015) and needs to rise to 95+% to meet SDG 11.6.1 (UN-Habitat, 2021), again doubling quantities to be managed. Collected wastes currently go to open dumping, and that needs to rise to 95+% controlled disposal. From Figure 2, that means the methane correction factor will increase from 0.4 to 1.0, a factor of 2.5 (controlled disposal sites do not yet have active gas control). So the methane emissions ignored by Hoy et al. (2023) from the 10% of global MSW currently generated by these countries can be expected (without intervention) to increase by a factor of 10 (2 × 2 × 2.5). So, although the article shows how the modelled emissions from 43 countries can be cut by around 50% thanks to the aggressive mitigation actions they discuss, the current 10% of current GHG emissions from the excluded majority of the lowest income countries can be expected to increase 10-fold, potentially becoming the dominant source of global waste emissions.

The increasing contribution over time of the Global South to total waste quantities and emissions demonstrated by this simple calculation is supported, for example, by World Bank forecasts (Hoornweg and Bhada-Tata, 2012; Kaza et al., 2018, 2021); by the earlier climate modelling study (Gómez-Sanabria et al., 2022); and by literature forecasts (Chen et al., 2020; Hoornweg et al., 2013, 2015).

### Unlocking he opportunity

This emphasises the urgent need, not only to improve MSWM in the Global South by meeting SDG 11.6.1; but to do that in a way that also mitigates methane emissions from controlled landfill sites. Measuring those emissions is clearly important; the Global South currently relies heavily on the IPCC estimation models (IPCC, 2006, 2019), often using default values for many variables. On-the-ground measurements are difficult and expensive; one option could be to use satellites, which is being used for monitoring global methane emissions, but remains challenging at the resolution required to pick out individual landfill sites (Gregory, 2024, personal communication).

What is required to move forward is a combination of three elements: extending waste collection to all; diverting as much as possible of the collected wastes from landfill; and moving beyond basic landfill control standards to capture and treat the landfill gas (Figure 2).

Diversion of waste from landfill means paying early attention to promote waste prevention and recycling, which not only reduces direct methane emissions but also provides significant indirect mitigation. Reducing food waste at source, as well as losses along the supply chain (SDG 12.3), will also help to end hunger (SDG 2). Increasing recycling rates (SDG 12.5) will divert waste from landfill and reduce both investment and operating costs. Separating the dominant organic fraction at source and collecting that separately (Pfaff-Simoneit, 2023) as a clean feedstock for organics recycling offers potential to improve soil (SDG 15.3), promote sustainable, local, small-scale food production (SDG 2.3 and 2.4), create jobs (SDG 8.5), generate biogas (SDG 7.2) and slash methane generation from landfilling the residual wastes (SDG 13). Increasing dry waste recycling rates can be achieved by building on the existing informal recycling sector and integrating them alongside the MSWM service (Aparcana, 2017; Cook et al., 2024; Scheinberg and Savain, 2015; Velis et al., 2012, 2022; Wilson, 2023). Separating wet organics at source, and/or collecting a dry recyclable fraction separately, enables the recyclers to work in cleaner and healthier conditions, leading to increased quantity and better quality of materials for recycling and improved, decent livelihoods (SDG 8.5).

Clean, source separated organic feedstock is critical to organics recycling, as it allows use of compost or anaerobic digestate for food crops, or indeed direct feeding to animals (Boumans et al., 2022) or breeding insects such as black soldier flies as a source of protein or fertiliser (Dortmans et al., 2021; Kim et al., 2021; ur Rehman et al., 2023). Such ‘prevention’ of contamination is at the top of ISWA’s ‘contaminant management hierarchy’ (Gilbert and Ricci-Jürgensen, 2023), and significantly reduces the potential for, and concentration of, contaminants such as plastics, chemicals or heavy metals. Municipalities in the Global South must be wary of anyone selling technologies that can ‘magically’ separate the organic fraction cleanly from mixed waste – any product derived from mixed waste is at best a ‘compost-like’ or ‘bio-stabilised’ material, which should be destined for use as municipal landscaping (e.g. alongside roads) or as soil cover on a landfill, and not be used for food crops (Cao et al., 2023; Gilbert and Ricci-Jürgensen, 2023; Saveyn and Eder, 2013).

Even if much waste is separated at source and diverted to both organics and dry waste recycling, there will be residual waste that requires controlled disposal to meet SDG 11.6.1, with resulting methane generation. Getting this far would be a major achievement for cities in the Global South, and beyond the affordability of many. Demanding that cities should only invest in relatively high-technology, full control (ESM) landfill sites, which generally need to be very large to achieve economies of scale, in order to control landfill gas (and leachate) to ‘modern’ standards, would generally have the perverse result of allowing open dumping to continue in most secondary cities in low- and lower-middle income countries as there is no practical and affordable alternative. Innovation and research is required in governance, service delivery and appropriate technologies to allow cities to upgrade or replace existing uncontrolled sites, both to meet SDG 11.6.1 and to move beyond basic operational control, at least to improved control by collecting and flaring landfill gas (Figure 2) (Wilson, 2023).

The genuine challenge of establishing affordable and environmentally acceptable landfill again opens the door to unscrupulous salespeople selling alternative ‘more advanced’ technologies, often energy-from-waste, to hard-pressed Mayors. Such technologies can make sense as part of an institutionally mature, well-functioning MSWM system which already separates waste at source and meets SDG 11.6.1, and where the city can afford to move on to the next steps. However, when a city is still struggling to develop a basic MSWM and recycling system, many of these technologies can be a dangerous distraction (Wilson, 2023). Several decision making guides are available for Mayors and their advisers, from GIZ (2017), UNEP (Liu and Nishiyama, 2020; UNEP IETC, 2019), World Bank (Kaza and Bhada-Tata, 2018) and US EPA (2020).

### The role of climate finance

Although the benefits of MSWM are both local and global, the costs are typically considered to be a local responsibility. The problem is that the level of user fees required to cover even the costs of basic services to meet SDG 11.6.1 (95+% mixed waste collection and controlled disposal) are unaffordable in many towns and cities in low- and middle-income countries (Scheinberg et al., 2010; Soos et al., 2015). To maximise the climate benefits, actions beyond SDG 11.6.1 are needed, to divert waste from landfill through separation at source to increase both organics and dry waste recycling, and to collect and control landfill gas. Local cost recovery via user fees is necessary, but needs to be complemented with income from extended producer responsibility (EPR) schemes, where the supply chain who place products or packaging on the market bear the full costs of collecting, recycling and properly disposing of those as part of MSW. EPR needs to be designed and implemented on a global or regional rather than a national basis, so that finance reaches the (smaller) lowest income countries who most need it.

Also, the international community needs to recognise the global benefits by targeting both climate finance, and ‘plastics finance’ under the new plastics treaty, to support the transition to sustainable MSW management practices (Wilson, 2023).

Official development finance first supported MSWM projects in the 1970s, but soon found that sustainable solutions required more than the capital costs of equipment (Arlosoroff, 1991; Wilson, 2023). This led to the development of the integrated sustainable waste management framework in the 1990s, which emphasises the need to consider all the technical aspects – collection, treatment and disposal and the 3Rs; and all the governance aspects – the stakeholders including the users and the service providers, financial sustainability, the national policy framework and local institutional coherence (Scheinberg et al., 2010; Schübeler, 1996; Van de Klundert and Anschütz, 2001; Wilson et al., 2013). Local capacity building is an essential component of most initiatives. There are lots of cautionary tales about failed projects, including full control landfills reverting to open dumps because the city could not afford the operating costs (Devadoss et al., 2021; Rouse, 2006). The old clean development mechanism (CDM) under the Kyoto Protocol was used to provide an income stream from utilisation of landfill gas, which provided a powerful incentive for cash-strapped cities to fund proper operation of their new landfill sites (Dulac, 2010). CDM has been replaced by Article 6 of the Paris Agreement, but this has yet to become fully operational (UNFCCC, n.d.).

What is needed now is a proper ‘joined-up’ approach, where all the different stakeholders – local and national, international development, climate change, plastics pollution, waste producers, the waste and resource sectors, non-governmental organisations (NGOs) and service users (citizens) – work together to enable win–win–win solutions across multiple SDGs (Wilson, 2023). Climate finance has a critical role to play, but does need to be designed to accommodate some specific characteristics of sustainable waste and resource management projects. These always go beyond just equipment. The waste needs to be collected, and preferably separated at source. Many recycling (and organic waste diversion) projects are community based. Project revenues will never cover costs – and recycling revenues should accrue to the informal (private) recyclers not to ‘the project’. Even controlled or ESM landfill projects are often in the mid $5–$95 million range that is less interesting to development agencies. Funding needs to reach communities, NGOs, municipalities and regional governments – this constrains development agencies who must work via national governments. It is important to complement traditional investment-led, top down official development assistance with people-focused, bottom up approaches (Practical Action, 2021; Wilson, 2023).

The cross-cutting nature of the benefits can appear to conflict with ‘additionality’, the criterion that projects need to provide GHG mitigation benefits that would not have happened otherwise. Calculating and monitoring the mitigation achieved is challenging, which can translate into high transaction costs for accreditation and verification (Wilson, 2023).

## Conclusions and call to action

IPCC recommends the integration of measures for better waste and resource management as a core part of planning for climate mitigation. However, that ‘*only 3% of global GHG emissions come from the waste sector*’ still influences perceptions. Global efforts to improve landfill control standards since the 1970s, and to divert organic waste from landfills since the 1990s, have steadily reduced direct emissions. But end-of-pipe SWM has also been evolving into more circular waste and resource management, with very significant indirect carbon savings from the 3Rs/9Rs (circular economy) which are attributed in the IPCC accounting conventions elsewhere in the economy.

This article has examined the evidence and compiled best estimates of the direct emissions and indirect savings in four categories, as summarised in the infographic Figure 1. Measurement is difficult and uncertain, and the system boundary is a bit ‘fuzzy’, but one can still, using IPCC terminology, have *high confidence* that the overall contribution of better waste and resource management to the mitigation of climate heating is significant. Whether that number is 15%, 20%, 25% or some other number, it does not affect the conclusion: the contribution is significant, and action is needed NOW to ensure that better waste and resource management, and improved resource efficiency, are included as a core part of every country’s climate mitigation plan / NDC.

Even the most advanced high-income countries still have much yet-to-be-tapped potential for climate mitigation from the 3Rs/9Rs. But much of the Global South still faces a global waste emergency; some 2.7 people worldwide do not have access to a MSW collection service, and 40% of collected MSW worldwide ends up in uncontrolled disposal which often includes open burning. Cities and countries often struggle to move towards the SDG 11.6.1 target of extending collection services and controlled disposal to 95+% of the population, which for many is simply unaffordable. The financial costs of better MSWM are local, but the benefits are both local – to public health and the environment; and global – to climate mitigation and preventing plastics pollution and plastics entering the oceans. So, addressing the global waste emergency is not only a local responsibility but also a global imperative.

However, meeting SDG 11.6.1 is not enough. Collecting and disposing all MSW in controlled landfill sites is a key step forward; in carbon terms it will eliminate open burning and black carbon, but will also bring an impact in terms of methane emissions (Figure 2). Simply extrapolating forward, MSW management meeting SDG 11.6.1 would take the smaller lowest income countries, that today generate only 10% of MSW worldwide, into the position of dominating global GHG emissions from the waste sector. What is needed is a more integrated approach, moving early to divert waste from landfill by separation at source, collecting separately a clean organic fraction for organics recycling and a clean, dry recycling fraction to enable the existing informal recycling sector to work in cleaner conditions, increase recycling rates and earn a better livelihood. This multifaceted approach tackles all four quadrants in Figure 1, making it a comprehensive strategy to combat the global waste emergency and move towards a more sustainable future.

Reaching SDG 11.6.1, and going beyond it to realise the significant climate mitigation benefits, makes it essential that climate finance should be designed specifically to target better MSWM in the lowest income countries; and that the roll-out of EPR should be on a global rather than a national basis so that it reaches also the (smaller) countries who need the financial support the most. A higher proportion of official development finance needs to be directed to enhance MSWM; UNEP and ISWA (2015) called for that to be increased from 0.3% to 3% per annum for a 15-year period.

Moving to the more integrated approach advocated here will take time; significant capacity building will be required; innovative approaches need to be developed and demonstrated at city level; and the resulting GHG mitigation needs to be integrated within NDCs and showcased. In the interim period, a complementary programme is also needed, to help unserved communities to help themselves, using a ‘bottom-up’, people-centred, service-led approach (Practical Action, 2021). However, the need to extend MSWM to all is mandatory; we cannot allow an insistence on an ‘ideal’ solution (such as the more integrated approach advocated here) stand in the way of a step by step approach, using simple, affordable and locally appropriate solutions.

We strongly urge global leaders to integrate sound waste and resource management into their climate mitigation plans / NDCs; and to ensure that international financial support is available to and reaches the countries that need it most. Alongside significant potential for climate mitigation at a reasonable cost, such actions will also facilitate equitable progress across a broad range of the SDGs.
